# Pharmacological Interventions for Opioid-Induced Hyperalgesia: A Scoping Review of Preclinical Trials

**DOI:** 10.3390/jcm11237060

**Published:** 2022-11-29

**Authors:** Mia Elena Koponen, Patrice Forget

**Affiliations:** 1 Neuroscience with Psychology, School of Medicine, Medical Sciences and Nutrition, University of Aberdeen, Aberdeen AB25 2ZD, UK; 2 Epidemiology Group, Institute of Applied Health Sciences, School of Medicine, Medical Sciences and Nutrition, University of Aberdeen, Aberdeen AB25 2ZD, UK; 3 Department of Anaesthesia, National Health Service (NHS) Grampian, Aberdeen AB25 2ZN, UK; 4 Pain and Opioids after Surgery (PANDOS) European Society of Anaesthesiology and Intensive Care (ESAIC) Research Group, 1000 Brussels, Belgium

**Keywords:** opioid-induced hyperalgesia, pain, remifentanil, fentanyl, morphine

## Abstract

Background: Opioid analgesics are the most effective pharmacological agents for moderate and severe pain. However, opioid use has several limitations such as opioid-induced hyperalgesia (OIH), which refers to the increased pain sensitivity that occurs once analgesia wears off after opioid administration. Several pharmacological interventions have been suggested for OIH, but the current literature does not provide guidelines on which interventions are the most effective and whether they differ depending on the opioid that induces hyperalgesia. This scoping review aimed to identify and describe all the preclinical trials investigating pharmacological interventions for OIH caused by remifentanil, fentanyl, or morphine as the first step towards evaluating whether the most effective OIH interventions are different for different opioids. Methods: Electronic database searches were carried out in Embase, PubMed, and Web of Science. Detailed data extraction was conducted on the eligible trials. Results: 72 trials were eligible for the review. Of these, 27 trials investigated remifentanil, 14 trials investigated fentanyl, and 31 trials investigated morphine. A total of 82 interventions were identified. The most studied interventions were ketamine (eight trials) and gabapentin (four trials). The majority of the interventions were studied in only one trial. The most common mechanism suggested for the interventions was inhibition of N-methyl-D-aspartate (NMDA) receptors. Conclusion: This scoping review identified plenty of preclinical trials investigating pharmacological interventions for OIH. Using the current literature, it is not possible to directly compare the effectiveness of the interventions. Hence, to identify the most effective interventions for each opioid, the interventions must be indirectly compared in a meta-analysis.

## 1. Introduction

Opioid analgesics are the most effective pharmacological agents for moderate and severe pain [1]. However, opioid use has several limitations such as the build-up of tolerance and a high risk for the development of addiction. One of the lesser-known limitations is opioid-induced hyperalgesia (OIH), which refers to the increased pain sensitivity that occurs once analgesia wears off after opioid administration [2]. OIH is commonly measured by quantitative sensory testing (QST), where mechanical or thermal stimuli are used to assess the subject’s pain threshold [3]. OIH has been mostly studied with surgical patients that have been administered remifentanil [4]. A few trials have observed OIH in chronic pain patients and addiction patients, but it is still unclear whether OIH is clinically significant in non-surgical patient groups [5,6]. To improve pain management with opioids, plenty of research has been carried out to identify interventions for OIH. Most of the interventions are pharmacological, but non-pharmacological interventions have also been investigated such as exercise [7], polyamine-deficient diet [8], and electroacupuncture therapy [9]. Current reviews of the pharmacological OIH interventions are qualitative and lack statistical analysis of effectiveness [10,11]. Additionally, there are two unpublished reviews registered in PROSPERO that aim to rank the effectiveness of pharmacological interventions for remifentanil-induced hyperalgesia (RIH) in surgical patients [12,13]. According to our preliminary searches, not enough randomised controlled trials (RCTs) have been carried out in other patient groups or using other opioids to evaluate the effectiveness of different interventions in these perspectives. Therefore, the current literature has a significant knowledge gap, as it is not known whether OIH in different contexts or caused by different opioids is attenuated most effectively by the same interventions. According to Heinl et al. [14], fentanyl-induced hyperalgesia (FIH) and morphine-induced hyperalgesia (MIH) occur via a distinct mechanism to RIH, which suggests that their most effective interventions may differ. Preliminary searches have shown that several preclinical trials have been conducted to investigate pharmacological interventions for OIH caused by remifentanil, fentanyl, or morphine. Hence, this scoping review aimed to identify all the preclinical trials investigating pharmacological interventions for OIH caused by remifentanil, fentanyl, or morphine as the first step towards evaluating whether the most effective OIH interventions are different for different opioids.

## 2. Aims

The aim of this scoping review was to identify and describe all the preclinical trials investigating pharmacological interventions for OIH caused by remifentanil, fentanyl, or morphine. Analysis of these trials will be carried out as the first step towards evaluating whether the most effective OIH interventions are different for different opioids. Hence, this review will qualitatively review the trials to (1) map the existing literature, (2) describe the trial characteristics, (3) identify gaps and limitations in the current research, and (4) make recommendations for future trials.

## 3. Methods

### 3.1. Protocol

This scoping review was planned according to the Preferred Reporting Items for Systematic Reviews and Meta-analysis Extension for Scoping Reviews (PRISMA-ScR) checklist developed by Tricco et al. [15]. See Appendix A for a summary of the checklist. A protocol to fulfil the requirements was planned as the first step of the review. The protocol included (1) preliminary searches into OIH intervention research to identify a knowledge gap and a suitable research question, (2) defining the eligibility criteria for including and excluding trials, (3) electronic database searches to gather potentially relevant research, (4) selecting eligible articles, (5) data extraction and description of the trials, and (6) summarising the findings and providing recommendations for future research. It should be noted that the protocol had a few minor deviations from the PRISMA-ScR checklist. The checklist includes a step for “review registration”, but since scoping reviews cannot be registered in the PROSPERO database, this step was skipped. Similarly, the methods subsection “critical appraisal of individual sources of evidence” and the results subsection “critical appraisal within sources of evidence” were skipped, as the assessment of the methodological quality of the included articles is not typically included in scoping reviews [16].

### 3.2. Eligibility Criteria

For a trial to be eligible in the review, it had to (1) investigate pharmacological intervention(s) for OIH, (2) investigate OIH caused by remifentanil, fentanyl, or morphine, (3) use an in vivo animal model, (4) measure hyperalgesia via QST, (5) be an original full research paper, and (6) be written in English. Pharmacological interventions with all timings, frequencies, dosages and administration methods were included. No time period restrictions were set.

### 3.3. Information Sources

The databases were selected for the search according to Bramer et al.’s [17] analysis of the “optimal database combinations for literature searches in systematic reviews”. Hence, to identify potentially relevant trials, electronic database searches were carried out in Embase, PubMed, and Web of Science. Reference lists of the eligible articles were scanned to find trials that may have been missed in the search. All searches were carried out in January 2022.

### 3.4. Search

The search strategy was created according to Leenaars et al.’s [18] “step-by-step guide to systematically identify all relevant animal studies” that combines search items for the disease of interest, interventions, and animal trials. In this review, the disease of interest words included, for example, “opioid-induced hyperalgesia” and “remifentanil-induced hyperalgesia”. The intervention search items included generic intervention words such as “drug therapy” and “pharmacological intervention”, as well as specific intervention words identified during preliminary searches such as “ketamine”. To identify all the available animal trials, the most recently developed animal filters were used [19]. The full search strategies for each database are shown in Appendix B.

### 3.5. Selection of Sources of Evidence

The articles obtained in the final search of each database were exported to EndNote. Duplicate articles were removed and the titles and abstracts were checked for eligibility. Full-text articles were evaluated in the next step if the eligibility was unclear. The process was repeated for the articles identified from the references of eligible articles.

### 3.6. Data Extraction

Data from eligible trials were charted onto a Microsoft Excel sheet. Data items extracted included the following categories: general, opioid, intervention, study design, QST, and animal model. The general information included the first author and year of publication. The type of opioid investigated was used to place the trial in the right category, and the dose, administration method, and regimen type were noted. The opioid regimen was categorised as “acute” if the opioid was given for less than 1 day and “chronic” if the opioid was given for several days. Intervention data items included the pharmacological agent used and its dose, administration method, administration time in comparison to opioid administration, and mechanism to attenuate OIH. In addition, intervention effectiveness was noted for each experimental group. Intervention in an experimental group was categorised as “effective” if statistical difference to the opioid-only group could be shown at any point with any type of QST. For the study design, the type and size of experimental and control groups were extracted, as well as the experimental injury model used and the length of the trial. In the trial lengths, catheterization procedures or baseline days were not taken into account. QST data items were the type of QST used, units of measurement, type of behaviour recorded (e.g., withdrawal or vocalisation), body area used for QST, and timing of measurements compared to opioid administration. Data items extracted about the animal model used included the species, strain, and sex of the animals.

For simplicity and relevance, not all of the study groups discussed in the articles were recorded. Groups exposed to the intervention without an opioid were excluded, since the analgesic property or the lack of it does not determine the intervention’s antihyperalgesic property. Similarly, groups that were exposed to the injury model without an opioid were excluded, as the research question specifically focuses on OIH and not other types of hyperalgesia. Groups that only provided additional information on the mechanism of the intervention were not included in the study groups. However, the information was used in data extraction to classify the intervention under the right mechanism. If a trial used several control groups, only the most relevant was included. For example, if both saline only and vehicle only groups were used, the vehicle group was selected. Moreover, whether the opioid only group received saline or other control injections (compared to the opioid and intervention group) was not recorded. Likewise, additional control exposures in other groups were not recorded.

### 3.7. Synthesis of Results

**Figure 1:**
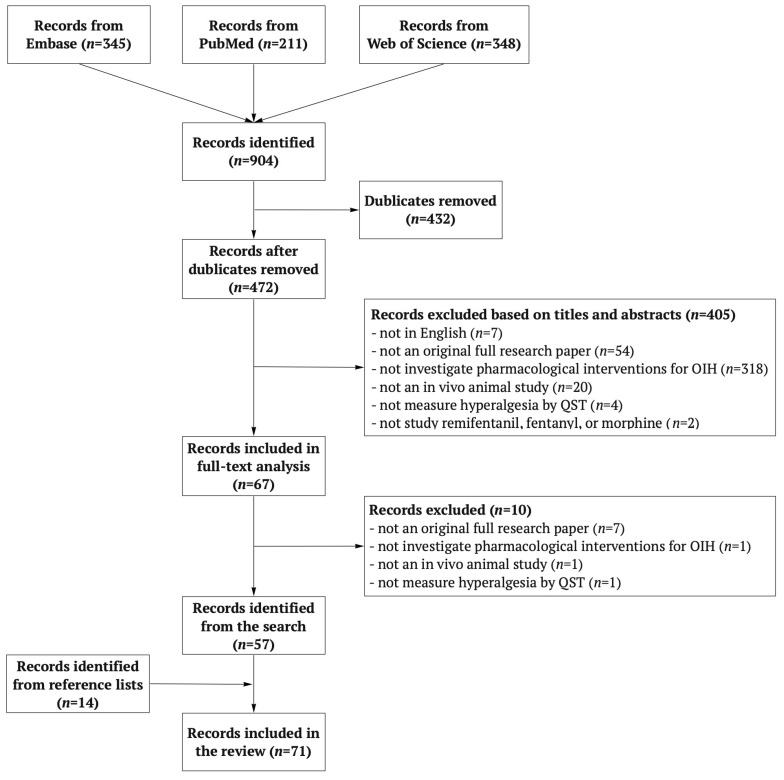
The process to identify eligible articles, in which 472 records were screened and 71 articles were included in the review.

The electronic database search results and the article screening process are summarised as a flow diagram in Figure 1. To be able to present the data extraction results without a supplementary materials section, three tables with only the main characteristics of each type of opioid trials were created (Table 1, Table 2 and Table 3). These included the first author, year of publication, intervention, study groups, intervention dose(s), administration method and injury model or opioid regimen used. The trial characteristics not included in these tables were described narratively. In addition, all the interventions investigated were listed separately for each opioid and are presented in Table 4. The interventions studied in more than one trial are summarised in Table 5. The mechanisms of the interventions are summarised in tables stating the intervention, its general class, the mechanism to attenuate OIH, and the mechanism group based on the shared pathway for OIH attenuation. Three intervention mechanism tables were created to separately represent the interventions studied for each type of opioid (Table 6, Table 7 and Table 8).

## 4. Results

### 4.1. Selection of Sources of Evidence

Electronic database searches in Embase, PubMed, and Web of Science provided 904 potentially relevant records (Figure 1). After the duplicates were removed, 472 records were screened for eligibility. Of these, 57 articles were deemed eligible and 14 further articles were identified by scanning the reference lists of eligible articles. A total of 71 articles were included in the review.

### 4.2. Characteristics of Sources of Evidence

The main characteristics of 72 trials investigating pharmacological interventions for OIH are presented in three tables in Section 4.3. Table 1 summarises 27 remifentanil trials, while 14 fentanyl trials are summarised in Table 2 and 31 morphine trials are summarised in Table 3. The trial by Wei and Wei [20] appears in two of the tables because the trial studied interventions for both FIH and MIH. The main characteristics presented are the first author, year of publication, intervention, study groups, intervention dose, administration method and injury model or opioid regimen used. The types of injections used for all of the interventions and opioids are marked by abbreviations: i.v. = intravenous, s.c. = subcutaneous, i.t. = intrathecal, i.p. = intraperitoneal, i.g. = intragastric. In the table for morphine trials, opioid regimen (acute or chronic) is presented instead of the injury models since only one trial used an injury model. On the other hand, in remifentanil and fentanyl trials, no chronic administration was used, so the injury models used are presented. In 23 of the morphine trials, the morphine regimen lasted for several days, and in 8 trials, the regimen consisted of only one morphine injection. In 22 remifentanil trials, a plantar incision injury model was used, while 5 trials did not include any injury model. In fentanyl trials, the plantar incision model was used in some of the experimental groups by Richebé et al. [21] and all experimental groups in the trial by Richebé et al. [22]. Some of the experimental groups in Richebé et al. [21] used a carrageenan injection model, which was also used by Bessière et al. [23], Van Elstraete et al. [24], and Richebé et al. [25]. Le Roy et al. [26] used a non-nociceptive environmental stress (NNES) model, which was also included in some of the experimental groups by Bessière et al. [23]. In morphine trials, Doyle et al. [27] were the only trial with an injury model and they used chronic constriction injury (CCI). Overall, most of the experimental groups marked as “not statistically different to the opioid only group” (#) were the lowest doses of the intervention that was successful with higher doses. Further comments about the interventions are included in Section 4.4. The data extraction items not included in the main characteristic tables are described below.

#### 4.2.1. Remifentanil Trials

In 23 of the remifentanil trials, male Sprague-Dawley rats were used as subjects. In addition, Aguado et al. [28] and Aguado et al. [29] used male Wistar rats, while Qi et al. [30] and Zhou et al. [31] used male Institute of Cancer Research (ICR) mice. The most common experimental group sizes were eight animals (12 trials) and six animals (5 trials). All of the trials used 6–12 animals per study group, apart from Cui et al. [32], who included 20 animals per study group. For QST, most trials used a mechanical and a thermal test. Cui et al. [33], Aguado et al. [28], and Aguado et al. [29] used only a mechanical test. Manual von Frey filaments were used in 13 of the trials, while another 13 trials used an electronic von Frey device. The Liu et al. [34] trial was the only one that used a dynamic plantar analgesiometer. The thermal tests used were a radiant heat paw withdrawal test (13 trials) and a hot plate test (11 trials). In all of the tests, the withdrawal of a hind paw was monitored. In 16 trials, the intervention was administered within a 30-min frame before remifentanil administration, and in 9 trials the intervention was coadministered with remifentanil. Lv et al. [35] administered betulinic acid for 7 days before remifentanil administration, while Li et al. [36] infused lithium chloride (LiCl) or thiadiazolidinone-8 (TDZD-8) for an hour before remifentanil infusion, and Liu et al. [34] administered N-acetyl-cysteine (NAC) 24 h after remifentanil. Most of the trials (16 trials) conducted measurements for 2 days. Cui et al. [33] conducted the shortest trial, which lasted 5 h, while Gu et al. [37] conducted the longest trial, lasting 21 days. The most common remifentanil administration regimens were 0.04 mg/kg subcutaneous infusion for 30 min (9 trials) or 1.0 μg/kg/min intravenous infusion for 60 min (6 trials).

#### 4.2.2. Fentanyl Trials

Male Sprague-Dawley rats were used in all of the fentanyl trials except in the Mert et al. [38] trial that used female Wistar rats. The most common experimental group sizes were 8 animals (five trials) and 10 animals (four trials). All of the trials used 6–12 animals per study group apart from Richebé et al. [25] and Van Elstraete et al. [39], which used 15 and 18 animals per study group respectively. Most of the trials used only one type of QST. However, Richebé et al. [22] used von Frey filaments and the Basile analgesimeter, and Li et al. [40] used von Frey filaments and a radiant heat paw withdrawal test. In total, eight of the trials used the Basile analgesimeter, where the hind paw pain threshold was marked by a vocalisation. A similar technique was used by the Van Elstraete et al. [41] trial using an analgesimeter. Four trials used von Frey filaments and three trials used a radiant heat paw withdrawal test, both of which recorded hind paw withdrawal. In six trials, the intervention was administered within a 30-min frame before fentanyl, and in four trials, the intervention was coadministered with fentanyl. Van Elstraete et al. [24] and Wei and Wei [20] administered gabapentin 30 min before or 5 h after fentanyl administration. Li et al. [40] administered KN93 6.5 h after fentanyl, whereas Kang et al. [42] administered ketorolac 24 h after fentanyl. Measurements were conducted for 1–7 days in 10 of the trials. Trials lasting for less than a day were conducted by Le Roy et al. [26] (5 h) and Laulin et al. [43] (6 h). Trials lasting for more than a week were conducted by Richebé et al. [22] (8 days) and Bessière et al. [23] (24–36 days). In 12 of the trials, the fentanyl regimen consisted of four 60–100 μg/kg subcutaneous injections in 15-min intervals. Le Roy et al. [26] used 50 ng/kg subcutaneous fentanyl injection and Mert et al. [38] used 3.5 μg intraplantar administration.

#### 4.2.3. Morphine Trials

In morphine trials, the animal models used were more heterogeneous than in remifentanil and fentanyl trials. Nine of the trials used male Sprague-Dawley rats. Female Sprague-Dawley rats were included in a few study groups in the trial by Doyle et al. [27]. Other animal models used in the morphine trials were: male Wistar rats, male ICR mice, male Swiss–Webster mice, male Swiss albino mice, male C57BL/6J mice, male CD-1 mice, and male Fischer 344 rats. The most common experimental group sizes were six animals (10 trials) and eight animals (7 trials). The majority of the trials used 6–12 animals per study group. Yet, Dunbar et al. [44] used 16–24 animals per study group and Chen et al. [45], Milne et al. [46], and Doyle et al. [27] had less than 4–5 animals in some study groups. For QST, 15 trials used only one type of test and 14 trials used two different tests. Raghavendra et al. [47], Dogrul et al. [48], and Tumati et al. [49] used three different tests. The most used tests were a radiant heat paw withdrawal test (14 trials), von Frey filaments (hind paw withdrawal, 11 trials), and warm water tail-flick test (8 trials). Sanna et al. [50] and Haleem and Nawas [51] recorded the licking of paws during a hot plate test. In addition, three trials monitored paw withdrawal using the hot plate test. Three further trials used a radiant heat tail-flick test. Dunbar et al. [52] and Corder et al. [53] monitored several types of withdrawal behaviours. Dunbar et al. [52] monitored head shaking, teeth chattering, squeaking, jumping, and urination in response to von Frey filaments while Corder et al. [53] monitored paw flinching, paw guarding, paw attending, and escape jumps on a hot plate. Ferrini et al. [54] recorded the number of vocalisations to subcutaneous morphine injections. In 20 trials the intervention was coadministered with morphine, and in 6 trials the intervention was administered within a 60-min frame before morphine. In 5 trials, the intervention was administered 1–4 days after morphine administration. In the trials, Tumati et al. [55], Tumati et al. [49], and Tumati et al. [56] small interfering RNA for rapidly accelerated fibrosarcoma 1 (Raf-1) or cyclic adenosine-monophosphate (cAMP)-dependent protein kinase A (PKA) was administered for 3 days before morphine. Chen et al. [45] administered ceftriaxone during the 3 days before morphine and during 4 days of morphine administration. Hua et al. [57] administered mesenchymal stem cells (MSC) either 1 day before, 7 days before, or 14 days after morphine administration. Lin et al. [58] administered heat shock protein 70 (HSP70) using an adenovirus 24 h before, 24 h after, and 72 h after the first morphine injection during a twice a day morphine injection regimen that lasted 6 days. The trial lengths of morphine trials were very variable. The most common lengths were 9 days (six trials) and 6 days (five trials). The shortest trial was conducted by Sanna et al. [50] in which they studied the effect of an µ opioid antagonist CTOP within 55 min. The longest trial was conducted by Doyle et al. [27] in which they studied the effect of fingolimod in the presence of CCI for 8 weeks. Acute morphine administration subcutaneously ranged from 0.1 μg/kg to 2.5 mg/kg and intrathecally from 0.05 ng to 10 mg/kg. Two of the acute regimen trials used 1 μg/kg intraperitoneal administration. Chronic morphine regimen ranged from 4 days to 28 days. The most common regimen lengths were 6 days (seven trials) and 7 days (six trials). The most common way to administer morphine was to give two 10 mg/kg subcutaneous injections in a day with 12-h intervals.

### 4.3. Results of Individual Sources of Evidence

Details of the 72 trials reviewed are shown in the tables below. Table 1 is a summary of the remifentanil trials, Table 2 is a summary of the fentanyl trials, and Table 3 is a summary of the morphine trials. 

**Table 1:** Main characteristics of remifentanil trials. Intervention, study groups, intervention dose, administration method and injury model used in 27 trials. #: experimental group was not statistically different compared to the opioid-only group. i.v.: intravenous. s.c.: subcutaneous. i.t.: intrathecal. i.p.: intraperitoneal. i.g.: intragastric. PI: plantar incision. N/A: no injury model was used.

Author and Year	Intervention	Study Groups	Dose and Administration	Injury
Cui et al. (2009) [33]	Lidocaine	(1) Propofol, (2) propofol and remifentanil, (3) propofol and lidocaine, (4) propofol and remifentanil and lidocaine	7.25 mg i.v. infusion for 120 min	PI
Gu et al. (2009) [59]	Ketamine	(1) Saline, (2) remifentanil, (3) remifentanil and ketamine	10 mg/kg s.c.	PI
Zheng et al. (2012) [60]	Dexmedetomidine	(1) Saline, (2) remifentanil, (3) dexmedetomidine 12.5 μg/kg and remifentanil, (4) dexmedetomidine 25 μg/kg and remifentanil, (5) dexmedetomidine 50 μg/kg and remifentanil	12.5 μg/kg, 25 μg/kg, 50 μg/kg s.c.	PI
Aguado et al. (2013) [28]	Naloxone	(1) Saline, (2) remifentanil, (3) remifentanil and naloxone	10 ng/kg i.v. bolus and 0.17 ng/kg/min i.v. infusion	N/A
Jiang et al. (2013) [61]	Ro 25-6981	(1) Saline, (2) remifentanil, (3) Ro 25-6981 200 μg and remifentanil, (4) Ro 25-6981 400 μg and remifentanil, (5) Ro 25-6981 800 μg and remifentanil	200 μg, 400 μg, 800 μg i.t.	PI
Li et al. (2013) [62]	TDZD-8	(1) Saline, (2) remifentanil, (3) remifentanil and TDZD-8	1.0 μg/kg/min i.v. infusion for 1 h	N/A
Yuan et al. (2013) [63]	TDZD-8	(1) Saline, (2) remifentanil, (3) TDZD-8 and remifentanil	1 mg/kg i.v. infusion	PI
Li et al. (2014) [36]	LiCl or TDZD-8	(1) Saline, (2) remifentanil, (3) LiCl and remifentanil, (4) TDZD-8 and remifentanil	LiCl: 100 mg/kg i.v. infusion. TDZD-8: 1 μg/kg i.v. infusion.	PI
Sun et al. (2014) [64]	JWH015	(1) Vehicle, (2) remifentanil, (3) JWH015 and remifentanil	10 μg i.v.	PI
Liu et al. (2014) [65]	Roscovitine	(1) Saline, (2) remifentanil, (3) roscovitine 25 μg and remifentanil, (4) roscovitine 50 μg and remifentanil, (5) roscovitine 100 μg and remifentanil	25 μg, 50 μg, 100 μg i.t.	PI
Zhang et al. (2014) [66]	Hydrogen-rich saline and/or Ro 25-6981	(1) Saline, (2) remifentanil, (3) HRS 10 mL/kg and remifentanil #, (4) HRS 2.5 mL/kg and remifentanil, (5) remifentanil and Ro 25-6981 5 μg #, (6) remifentanil and Ro 25-6981 10 μg, (7) remifentanil and Ro 25-6981 50 μg, (8) HRS 2.5 mL/kg and remifentanil and Ro 25-6981 5 μg	HRS: 10 mL/kg, 2.5 mL/kg i.p. Ro 25-6981: 5 μg, 10 μg, 50 μg i.t.	PI
Aguado et al. (2015) [29]	Amitriptyline or minocycline or maropitant	(1) Saline, (2) remifentanil, (3) amitriptyline and remifentanil #, (4) minocycline and remifentanil #, (5) maropitant and remifentanil #	Amitriptyline: 50 mg/kg i.p. Minocycline: 100 mg/kg i.p. Maropitant: 30 mg/kg i.p.	N/A
Jiang et al. (2015) [67]	KN93	(1) Saline, (2) remifentanil, (3) KN93 25 μg/kg and remifentanil #, (4) KN93 50 μg/kg and remifentanil, (5) KN93 100 μg/kg and remifentanil	25 μg/kg, 50 μg/kg, 100 μg/kg i.t.	PI
Sun et al. (2015) [68]	Magnesium or ketamine	(1) Saline, (2) remifentanil, (3) ketamine and remifentanil, (4) magnesium 100 μg and remifentanil, (5) magnesium 300 μg and remifentanil	Ketamine: 10 μg i.t. Magnesium: 100 μg, 300 μg i.t.	PI
Wang et al. (2015) [69]	Naltrindole	(1) Saline, (2) remifentanil, (3) naltrindole and remifentanil	10 μL of 30 nM i.t.	PI
Zhang et al. (2015) [70]	PHA-543613 and/or PNU-120596	(1) Saline, (2) remifentanil, (3) PHA 3 μg and remifentanil, (4) PHA 6 μg and remifentanil, (5) PHA 12 μg and remifentanil, (6) PHU 2 μg and remifentanil, (7) PHU 4 μg and remifentanil, (8) PHU 8 μg and remifentanil, (9) PHA 6 μg and PHU 4 μg and remifentanil	PHA-543613: 3 μg, 6 μg, 12 μg i.t. PNU-120596: 2 μg, 4 μg, 8 μg i.t.	PI
Cui et al. (2016) [32]	Lidocaine	(1) Experimentally naïve, (2) remifentanil, (3) lidocaine and remifentanil	200 mg/kg/min i.v. infusion	N/A
Gu et al. (2017) [37]	PNU-120596 or BDNF-sequester TrkB/Fc	(1) Saline, (2) remifentanil, (3) BDNF-sequester TrkB/Fc and remifentanil, (4) PNU-120596 and remifentanil	PNU-120596: 8 µg/kg i.t. BDNF-sequester TrkB/Fc: 5 µg i.t.	PI
Liu et al. (2017) [34]	N-acetyl-cysteine	(1) Saline, (2) remifentanil, (3) remifentanil and NAC 25 mg/kg, (4) remifentanil and NAC 75 mg/kg, (5) remifentanil and NAC 150 mg/kg	25 mg/kg, 75 mg/kg, 150 mg/kg i.p.	PI
Liu et al. (2017) [71]	Naltrindole	(1) Saline, (2) remifentanil, (3) remifentanil and naltrindole	10 μL of 30 nM i.t.	PI
Sun et al. (2017) [72]	Magnesium	(1) Remifentanil, (2) magnesium 100 μg and remifentanil, (3) magnesium 300 μg and remifentanil	100 μg, 300 μg i.t.	PI
Yuan et al. (2017) [63]	Dexmedetomidine	(1) Saline, (2) remifentanil, (3) dexmedetomidine and remifentanil	50 μg/kg s.c.	PI
Lv et al. (2018) [35]	Betulinic acid	(1) Vehicle, (2) remifentanil, (3) betulinic acid and remifentanil	25 mg/kg i.g. for 7 days	PI
Li et al. (2019) [73]	Anxa1(2-26)	(1) Saline, (2) remifentanil, (3) Anxa1(2-26) 5 μg and remifentanil #, (4) Anxa1(2-26) 50 μg and remifentanil, (5) Anxa1(2-26) 500 μg and remifentanil	5 μg, 50 μg, 500 μg i.t.	N/A
Gao et al. (2020) [74]	IWP-2 or Ro 25-6981	(1) Saline, (2) remifentanil, (3) IWP-2 60 μM and remifentanil #, (4) IWP-2 120 μM and remifentanil, (5) IWP-2 180 μM and remifentanil, (6) Ro 25-6981 and remifentanil	IWP-2: 60 μM, 120 μM, 180 μM i.t. In 10 μL. Ro25-6981: 1.5μg i.t.	PI
Qi et al. (2020) [30]	Ketamine and/or KN93	(1) Saline, (2) remifentanil, (3) ketamine 1.4 mg/kg and remifentanil, (4) ketamine 2.1 mg/kg and remifentanil, (5) ketamine 2.8 mg/kg and remifentanil, (6) KN93 50 μg/kg and remifentanil, (7) KN93 75 μg/kg and remifentanil, (8) KN93 100 μg/kg and remifentanil, (9) KN93 100 μg/kg and ketamine 2.1 mg/kg and remifentanil	Ketamine: 1.4 mg/kg, 2.1 mg/kg, 2.8 mg/kg s.c. infusion. KN93: 50 μg/kg, 75 μg/kg, 100 μg/kg i.t.	PI
Zhou et al. (2020) [31]	Dezocine	(1) Saline, (2) remifentanil, (3) dezocine 1.5 mg/kg and remifentanil #, (4) dezocine 3 mg/kg and remifentanil, (5) dezocine 6 mg/kg and remifentanil, (6) ketamine and remifentanil	Dezocine: 1.5 mg/kg, 3 mg/kg, 6 mg/kg s.c. infusion. Ketamine: 2.8 mg/kg s.c. infusion.	PI

**Table 2:** Main characteristics of fentanyl trials. Intervention, study groups, intervention dose, administration method, and injury model used in 14 trials. #: experimental group was not statistically different from the opioid-only group. s.c.: subcutaneous. i.t.: intrathecal. i.p.: intraperitoneal. PI: plantar incision. CI: carrageenan injection. S: non-nociceptive environmental stress. N/A: no injury model was used. *: injury model not used in all study groups.

Author & Year	Intervention	Study Groups	Dose & Administration	Injury
Célèrier et al. (2000) [75]	Ketamine	(1) Saline, (2) fentanyl, (3) ketamine and fentanyl	10 mg/kg s.c.	N/A
Kang et al. (2002) [42]	Ketorolac	(1) Fentanyl and 5 μg ketorolac #, (2) fentanyl and 15 μg ketorolac, (3) fentanyl and 50 μg ketorolac	5 μg, 15 μg or 50 μg i.t.	N/A
Liukin et al. (2002) [43]	Ketamine	(1) Saline, (2) fentanyl, (3) ketamine and fentanyl	10 mg/kg s.c.	N/A
Richebé et al. (2005) [21]	Nitrous oxide	(1) Saline, (2) fentanyl, (3) fentanyl and N2O 10%, (4) fentanyl and N2O 20%, (5) fentanyl and N2O 30%, (6) fentanyl and N2O 40%, (7) fentanyl and PI and N2O 50%, (8) fentanyl and CI and N2O 20% #, (9) fentanyl and CI and N2O 30%, (10) fentanyl and CI and N2O 40%, (11) fentanyl and CI and N2O 50%	10%, 20%, 30%, 40%, 50% inhalation for 4 h 15 min	PI * or CI *
Richebé et al. (2005) [22]	Ketamine	(1) Saline, (2) fentanyl, (3) ketamine and fentanyl	10 mg/kg s.c. three times with 5 h intervals	PI
Van Elstraete et al. (2006) [41]	Magnesium	(1) Saline, (2) fentanyl, (3) magnesium and fentanyl	100 mg/kg i.p.	N/A
Bessière et al. (2007) [23]	Nitrous oxide	(1) Saline, (2) fentanyl and CI+CI, (3) fentanyl and CI+CI and N_2_O, (4) fentanyl and CI+S, (5) fentanyl and CI+S and N2O	50% inhalation for 4 h 15 min	CI+CI * or CI+S *
Van Elstraete et al. (2008) [24]	Gabapentin	(1) Saline, (2) fentanyl, (3) fentanyl and gabapentin 30 mg/kg i.p. 30 min before first fentanyl dose #, (4) fentanyl and gabapentin 75 mg/kg i.p. 30 min before first fentanyl dose, (5) fentanyl and gabapentin 150 mg/kg i.p. 30 min before first fentanyl dose, (6) fentanyl and gabapentin 300 mg/kg i.p. 30 min before first fentanyl dose, (7) fentanyl and gabapentin 150 mg/kg i.p. 300 min after last fentanyl dose, (8) fentanyl and gabapentin 300 μg 30 min i.t. before first fentanyl dose, (9) CI and fentanyl and saline, (10) CI and fentanyl and gabapentin 150 mg/kg i.p. 30 min before first fentanyl dose	30 mg/kg, 75 mg/kg, 150 mg/kg, 300 mg/kg i.p. or 300 μg i.t.	CI *
Mert et al. (2009) [38]	Magnesium	(1) Saline, (2) fentanyl, (3) fentanyl and magnesium	2 mg intraplantar injection	N/A
Richebé et al. (2009) [25]	Sevoflurane	(1) Saline, (2) fentanyl, (3) sevoflurane 1% and fentanyl, (4) CI and fentanyl, (5) CI and sevoflurane 1% and fentanyl #, (6) CI and sevoflurane 1.5% and fentanyl #	1%, 1.5% inhalation for 4 h 30 min	CI *
Le Roy et al. (2011) [26]	BN2572	(1) Saline, (2) fentanyl, (3) BN2572 and fentanyl	0.3 mg/kg s.c.	S
Van Elstraete et al. (2011) [39]	Ketamine and/or gabapentin	(1) Ketamine and fentanyl, (2) gabapentin and fentanyl, (3) ketamine and gabapentin and fentanyl	Ketamine: 10–15 mg/kg s.c. Gabapentin: 270–330 300 mg/kg i.p. Ketamine-gabapentin combination: 3–6 mg/kg s.c. and 90–180 mg/kg i.p.	N/A
Wei and Wei (2012) [20]	Gabapentin	(1) Saline, (2) fentanyl, (3) gabapentin 25 mg/kg and fentanyl, (4) gabapentin 50 mg/kg and fentanyl	25 mg/kg, 50 mg/kg i.p.	N/A
Li et al. (2016) [40]	KN93	(1) Fentanyl, (2) fentanyl and KN92, (3) fentanyl and KN93 5 nmol, (4) fentanyl and KN93 7.5 nmol, (5) fentanyl and KN93 10 nmol	5–10 nmol microinjection to amygdala	N/A

**Table 3:** Main characteristics of morphine trials. Intervention, study groups, intervention dose, administration method, and opioid regimen (chronic/acute) used in 31 trials. #: experimental group was not statistically different from the opioid only group. i.v. = intravenous. s.c.: subcutaneous. i.t.: intrathecal. i.p.: intraperitoneal. i.g.: intragastric. C: chronic opioid regimen. A: acute opioid regimen. CCI: chronic constriction injury.

Author and Year	Intervention	Study Groups	Dose and Administration	Chronic/ Acute
Dunbar et al. (2000) [52]	Ibuprofen	(1) Saline, (2) morphine, (3) morphine and ibuprofen 136 nM, (4) morphine and ibuprofen 13.6 nM, (5) morphine and ibuprofen 1.36 nM #	136 nM, 13.6 nM, 1.36 nM in 10 μL bolus via spinal catheter	C
Crain and Shein (2001) [76]	Naltrexone	(1) Saline, (2) morphine 1 μg/kg, (3) naltrexone 1 ng/kg and morphine 1 μg/kg, (4) morphine 0.1 μg/kg, (5) naltrexone 1 pg/kg and morphine 0.1 μg/kg	1 ng/kg, 1 pg/kg injection	A
Raghavendra et al. (2004) [47]	Propentofylline	(1) Saline, (2) morphine, (3) morphine and propentofylline 1 μg, (4) morphine and propentofylline 10 μg	1 μg, 10 μg i.t. daily for 5 days	C
Dogrul et al. (2005) [48]	Amlodipine	(1) Saline, (2) morphine, (3) amlodipine and morphine	10 μg i.t. twice a day for 8 days	C
Van Elstraete et al. (2005) [77]	Ketamine	(1) Saline, (2) morphine, (2) ketamine and morphine	10 mg/kg s.c.	A
Juni et al. (2006) [78]	MK-801	(1) Placebo pellets and morphine, (2) placebo pellets and MK-801 and morphine	0.05 mg/kg MK-801 s.c.	C
Dunbar et al. (2007) [44]	Ketorolac	(1) Saline, (2) morphine, (3) morphine and ketorolac, (4) morphine and naloxone, (5) morphine and ketorolac and naloxone	5 mg/kg s.c. daily for 4 days	C
Esmaeili-Mahani et al. (2007) [79]	Nifedipine	(1) Vehicle, (2) morphine 1 μg/kg, (3) nifedipine 2 mg/kg and morphine 1 μg/kg, (4) morphine 0.01 μg/kg, (5) nifedipine 10 μg/kg and morphine 0.01 μg/kg	2 mg/kg i.p., 10 μg/kg i.t.	A
Tumati et al. (2008) [55]	Raf-1 siRNA	(1) Vehicle, (2) morphine, (3) morphine and non-targeting dsRNA, (4) morphine and Raf-1 siRNA	2 µg i.t. once a day for 3 days	C
Esmaeili-Mahani et al. (2010) [80]	Olive leaf extract	(1) Vehicle, (2) morphine, (3) morphine and OLE 6 mg/kg, (4) morphine and OLE 12 mg/kg	6 mg/kg, 12 mg/kg i.p.	A
Tumati et al. (2010) [49]	Raf-1 siRNA	(1) Vehicle, (2) morphine, (3) non-targeting dsRNA and morphine, (4) Raf-1 siRNA and morphine	2 µg i.t. once a day for 3 days	C
Gupta et al. (2011) [81]	Magnesium or dextromethorphan or d-serine	(1) Saline, (2) morphine, (3) morphine and magnesium 2 mg/kg, (4) morphine and magnesium 5 mg/kg, (5) morphine and dextromethorphan 2 mg/kg, (6) morphine and dextromethorphan 5 mg/kg, (7) morphine and dextromethorphan 10 mg/kg, (8) morphine and d-serine 2 mg/kg, (9) morphine and d-serine 5 mg/kg, (10) morphine and d-serine 10 mg/kg	Magnesium: 2 mg/kg, 5 mg/kg i.p. Dextromethorphan: 2 mg/kg, 5 mg/kg, 10 mg/kg i.p. D-serine: 2 mg/kg, 5 mg/kg, 10 mg/kg i.p.	A
Liang et al. (2011) [82]	Ondansetron	(1) Morphine, (2) morphine and ondansetron s.c., (3) morphine and ondansetron s.c. twice a day for 4 days, (4) morphine and ondansetron i.t., (5) morphine and ondansetron 1 μg peripheral injection #, (6) morphine and ondansetron 10 μg peripheral injection #	2 mg/kg s.c. or 1 mg/kg s.c. twice a day for 4 days or 1 μg i.t. or 1 μg, 10 μg peripheral hind paw injection	C
Tumati et al. (2011) [56]	PKA siRNA	(1) Vehicle, (2) morphine, (3) PKA siRNA and morphine	2 µg i.t. once a day for 3 days	C
Chen et al. (2012) [45]	Ceftriaxone	(1) Saline, (2) morphine, (3) ceftriaxone and morphine	200 mg/kg i.p. daily for 7 days	C
Tumati et al. (2012) [83]	L-732,138	(1) Saline, (2) morphine, (3) morphine and L-732,138	20 μg/5 μL i.t. twice a day for 6 days	C
Wei and Wei (2012) [20]	Gabapentin	(1) Saline, (2) morphine, (3) gabapentin 25 mg/kg and morphine, (4) gabapentin 50 mg/kg and morphine	25 mg/kg, 50 mg/kg i.p.	C
Xin et al. (2012) [84]	Melatonin	(1) Saline, (2) morphine, (3) morphine and melatonin 25 mg/kg #, (4) morphine and melatonin 50 mg/kg, (5) morphine and melatonin 100 mg/kg	25 mg/kg, 50 mg/kg, 100 mg/kg i.g. once a day for 7 days	C
Milne et al. (2013) [46]	Efaroxan or atipamezole or yohimbine	(1) Saline, (2) morphine, (3) morphine and (+) efaroxan, (4) morphine and (-) efaroxan #, (5) morphine and atipamezole, (6) morphine and yohimbine	Efaroxan: 1.3 ng i.t. Atipamezole: 0.08 ng i.t. Yohimbine: 0.02 ng i.t.	A
Orrù et al. (2014) [85]	Withania somnifera root extract	(1) Saline, (2) morphine, (3) WSE and morphine	100 mg/kg i.p.	A
Li et al. (2014) [86]	Re or Rg1 or Rb1 ginsenosides	(1) Saline, (2) morphine, (3) morphine and Re 100 mg/kg #, (4) morphine and Re 200 mg/kg #, (5) morphine and Re 300 mg/kg, (6) morphine and Rg1 100 mg/kg #, (7) morphine and Rg1 200 mg/kg #, (8) morphine and Rg1 300 mg/kg #, (9) morphine and 100 mg/kg Rb1 #, (10) morphine and Rg1 200 mg/kg #, (11) morphine and Rg1 300 mg/kg #	100 mg/kg, 200 mg/kg, 300 mg/kg i.g. twice a day for 2 days	C
Sanna et al. (2015) [50]	CTOP or PD98059	(1) Saline, (2) morphine, (3) morphine and CTOP, (4) PD98059 and morphine	PD98059: 20 µg i.t. CTOP: 0.1 mg i.t.	A
Song et al. (2015) [87]	Melatonin	(1) Saline, (2) morphine, (3) morphine and melatonin	10 mg/kg i.p.	C
Hu et al. (2016) [88]	PLGA curcumin nanoformulation or unformulated curcumin	(1) Vehicle i.t., (2) morphine, (3) morphine and unformulated curcumin [6], (4) vehicle oral, (5) morphine and PLGA curcumin 2 mg/kg #, (6) morphine and PLGA curcumin 6 mg/kg, (7) morphine and PLGA curcumin 20 mg/kg	Unformulated curcumin: 30 μg i.t. PLGA curcumin: 2 mg/kg, 6 mg/kg, 20 mg/kg oral administration.	C
Hua et al. (2016) [57]	MSC	(1) Saline, (2) morphine, (3) MSC i.t. 1 day before and morphine, (4) MSC i.v. 1 day before and morphine, (5) MSC i.t. 7 day before and morphine, (6) MSC i.v. 7 day before and morphine, (7) morphine and MSC i.t. on day 14, (8) morphine and MSC i.v. on day 14	0.5 million MSCs i.t. or 0.5 million MSCs i.v.	C
Corder et al. (2017) [53]	Methylnaltrexone bromide	(1) Saline, (2) morphine, (3) morphine and methylnaltrexone bromide	10 mg/kg s.c. once a day for 7 days	C
Ferrini et al. (2017) [54]	CLP257 or CLP290	(1) Saline, (2) morphine, (3) morphine and CLP290, (4) morphine and CLP257 on day 9, (5) morphine and CLP257 on day 7 and 8	CLP290: 100 mg/kg orally twice a day for 7 days. CLP257: 100 mg/kg i.p. on day 7 and 8 or only day 9	C
Haleem and Nawas (2017) [51]	Buspirone	(1) Saline, (2) morphine, (3) morphine and buspirone 1 mg/kg, (4) morphine and buspirone 2 mg/kg	1 mg/kg or 2 mg/kg i.p.	C
Datta et al. (2020) [89]	DAMGO or GAT211	(1) Saline, (2) morphine, (3) morphine and DAMGO 0.03 μg, (4) morphine and DAMGO 0.1 μg, (5) morphine and DAMGO 0.3 μg, (6) morphine and DAMGO 1 μg, (7) morphine and GAT211 1 μg #, (8) morphine and GAT211 1 μg #, (9) morphine and GAT211 5 μg #, (10) morphine and GAT211 10 μg #, (11) morphine and GAT211 20 μg #	DAMGO: 0.03 μg, 0.1 μg, 0.3 μg, 1 μg. GAT211: 1 μg, 5 μg, 10 μg, 20 μg	C
Doyle et al. (2020) [27]	W146 or JTE-013 or CAY10444 or NIBR-14 or SEW2871 or S1pr1 siRNA or NIBR-15 or fingolimod or ponesimod	(1) Saline, (2) morphine, (3) morphine and W140, (4) morphine and W146 0.2 nmol/day, (5) morphine and W146 0.7 nmol/day, (6) morphine and W146 2 nmol/day, (7) morphine and JTE-013 #, (8) morphine and CAY1044 #, (9) morphine and NIBR-14 i.t. 3 nmol/day, (10) morphine and SEW2871 2 nmol/day #, (11) morphine and SEW2871 20 mg/kg/day #, (12) morphine and non-targeting siRNA, (13) morphine and S1pr1 siRNA, (14) morphine and oral NIBR-14 0.3 mg/kg/day #, (15) morphine and oral NIBR-14 1 mg/kg/day, (16) morphine and oral NIBR-14 3 mg/kg/day, (17) morphine and oral NIBR-15 3 mg/kg/day, (18) morphine and fingolimod 0.03 mg/kg/day #, (19) morphine and fingolimod 0.01 mg/kg/day, (20) morphine and fingolimod 0.1 mg/kg/day male rats, (21) morphine and fingolimod 0.1 mg/kg/day female rats, (22) morphine and ponesimod, (23) CCI and saline and vehicle, (24) CCI and morphine and vehicle, 25) CCI and morphine and fingolimod	W146: 0.2 nmol/day, 0.7 nmol/day, 2 nmol/day. JTE-013: 2 nmol/day. CAY1044: 2 nmol/day. NIBR-14: 3 nmol/day i.t., 0.3 mg/kg/day oral, 1 mg/kg/day oral, 3 mg/kg/day. NIBR-15: 3 mg/kg/day oral. SEW2871: 2 nmol/day i.t., 20 mg/kg/day i.p. S1pr1 siRNA: 2 μg i.t. Fingolimod: 0.03 mg/kg/day oral, 0.01 mg/kg/day oral, 0.1 mg/kg/day oral. Ponesimod: 3 mg/kg/day.	C
Lin et al. (2020) [58]	TUDCA or AEBSF or 4μ8C or salubrinal or adenovirus-HSP70 or glibenclamide	(1) Vehicle, (2) morphine, (3) TUDCA and morphine, (4) AEBSF and morphine, (5) 4μ8C and morphine, (6) salubrinal and morphine #, (7) adenovirus-HSP70 and morphine, (8) glibenclamide 0.08 μg and morphine, (9) glibenclamide 0.4 μg and morphine, (10) glibenclamide 2 μg and morphine	TUDCA: 100 μg/10 μL i.t. Glibenclamide or TUDCA or AEBSF or 4μ8C or salubrinal or adenovirus-HSP70. Glibenclamide: 0.08 μg, 0.4 μg, 2 μg.	C

### 4.4. Synthesis of Results

From the 72 trials eligible for the review, 82 different interventions were identified. Remifentanil trials investigated 27 interventions, fentanyl trials investigated 9 interventions, and morphine trials investigated 52 interventions. Table 4 lists all the interventions studied for each type of opioid. If an intervention was different from the control in at least one of the investigated doses it is listed as “effective”. The number of trials an intervention was studied in is marked by an X and a number following the intervention. If an intervention was studied for several opioids, it is marked by an asterisk (*). The interventions that were studied most are placed at the top of the lists, while the ineffective interventions are placed at the bottom. Combination interventions were counted as separate interventions from their single interventions and were placed on the list before single interventions. Otherwise, the interventions are presented in arbitrary order.

Ketamine was the most studied intervention in the remifentanil and fentanyl trials. In morphine trials, the most studied interventions were melatonin and Raf-1 selective siRNA. In the fentanyl trials, the only combination intervention studied was ketamine and gabapentin (Van Elstraete et al. [39]) while no combinations were studied in the morphine trials. In the remifentanil trials, three combinations were studied: hydrogen-rich saline and Ro 25-6981 (Zhang et al. [66]), PHA-543613 and PNU-120596 (Zhang et al. [70]), and ketamine and KN93 (Qi et al. [30]). In the fentanyl trials, all interventions demonstrated potential in attenuating OIH. In the remifentanil trials, amitriptyline, minocycline, and maropitant studied by Aguado et al. [29] were the only interventions that were not found effective at all. In the morphine trials, six interventions were found unsuccessful: Rg1 ginsenoside, Rb1 ginsenoside, GAT211, JTE-013, CAY10444, and SEW2871.

**Table 4:** Interventions studied for each type of opioid. Remifentanil trials investigated 27 interventions, fentanyl trials investigated 9 interventions, and morphine trials investigated 52 interventions. An intervention is listed as “ineffective” if none of the tested doses were different from the control. *: the intervention is studied for the other opioids too.

Remifentanil (27)		Fentanyl (9)		Morphine (52)	
Effective	Ineffective	Effective	Ineffective	Effective	Ineffective
Ketamine ×3 * Ro 25-6981 ×3 TDZD-8 ×3 KN93 x2 * Lidocaine ×2 Dexmedetomidine ×2 Naltrindole ×2 PNU-120596 ×2 Hydrogen-rich saline and Ro 25-6981 PHA-543613 and PNU-120596 Ketamine and KN93 Magnesium * Naloxone LiCl JWH015 Roscovitine Hydrogen rich saline PHA-543613 BDNF-sequester TrkB/Fc N-acetyl-cysteine Betulinic acid Anxa1(2-26) IWP-2 Dezocine	Amitriptyline Minocycline Maropitant	Ketamine ×4 * Gabapentin ×3 * Magnesium ×2 * Nitrous oxide ×2 Ketorolac * KN93 * Ketamine and gabapentin BN2572 Sevoflurane	-	Raf-1 siRNA ×2 Melatonin ×2 Ketamine * Ketorolac * Gabapentin * Ibuprofen Naltrexone Methylnaltrexone bromide Propentofylline Amlodipine MK-801 Nifedipine Olive leaf extract Magnesium Dextromethorphan D-serine Ondansetron PKA siRNA Ceftriaxone L-732,138 Efaroxan Atipamezole Yohimbine Withania somnifera root extract CTOP PD98059 PLGA curcumin Curcumin Mesenchymal stem cells CLP257 CLP290 Buspirone DAMGO W146 NIBR-14 S1pr1 siRNA NIBR-15 Fingolimod Ponesimod TUDCA AEBSF 4μ8C Salubrinal Adenovirus-HSP70 Glibenclamide Re ginsenoside	Rg1 ginsenoside Rb1 ginsenoside GAT211 JTE-013 CAY10444 SEW2871

Interventions studied in more than one trial are presented in Table 5. Only 14 of the 82 interventions (17%) were studied more than once. Overall, ketamine was the most-investigated intervention, as it was included in eight trials. The next-most-studied intervention was gabapentin, which was included in four trials.

**Table 5:** Interventions investigated overall in more than one trial. More than one trial was devoted to studying 14 of 82 interventions. Ketamine was the most-studied intervention.

In 8 Trials	In 4 Trials	In 3 Trials	In 2 Trials
Ketamine	Gabapentin	Magnesium Ro 25-6981 TDZD-8 KN93	Lidocaine Dexmedetomidine Naltrindole PNU-120596 Nitrous oxide Ketorolac Raf-1 siRNA Melatonin

The mechanisms of the interventions used in the remifentanil trials are summarised in Table 6, while Table 7 and Table 8 summarise the mechanisms of the interventions used in the fentanyl and morphine trials, respectively. These tables display each intervention’s general class, the suggested mechanism for OIH attenuation, and the mechanism group based on the shared pathway for OIH attenuation. The interventions with similar mechanisms have been placed close to each other, but otherwise the content is presented in an arbitrary order. Compartments in the table are merged when interventions or mechanisms belong to the same category. Combination interventions are separated into their individual interventions, and their possible mechanisms for additive or synergic effects were not included in the analysis.

Table 6 presents the 24 interventions studied in remifentanil trials. The largest mechanism group is N-methyl-D-aspartate receptor (NMDAR) inhibition that includes NMDAR block, NMDAR antagonism, NMDAR expression inhibition, NMDAR phosphorylation inhibition, NMDAR trafficking inhibition as well as calcium/calmodulin-dependent protein kinase II (CaMKII) inhibition and CaMKII phosphorylation inhibition, which influence NMDARs. Six other interventions include NMDAR inhibition in their mechanism group but also influence OIH via other mechanisms. Other most common mechanisms include proinflammatory cytokine reduction (five trials), α-amino-3-hydroxy-5-methyl-4-isoxazolepropionic acid receptor (AMPAR) inhibition (three interventions), and inhibition of glial cells (three trials).

**Table 6:** Mechanisms of the interventions in remifentanil trials. General class of 24 interventions, OIH attenuation mechanism, and mechanism group based on the shared pathway for OIH attenuation.

Intervention	General Class	OIH Attenuation Mechanism	Mechanism Group
Ketamine	NMDAR blocker	NMDAR block	NMDAR inhibition
Magnesium			
Ro 25–6981	NR2B antagonist	NMDAR antagonism	
Amitriptyline	Tricyclic antidepressant		
Naltrindole	Selective DOR inhibitor	NMDAR antagonism, NMDAR expression and trafficking inhibition	
Dexmedetomidine	α2-adrenergic agonist	NMDAR antagonism, NMDAR phosphorylation inhibition, NMDAR expression and trafficking inhibition	
IWP-2	Wnt3a inhibitor	NMDAR expression inhibition	
Hydrogen-rich saline	Superoxide remover	NMDAR expression and trafficking inhibition	
KN93	CaMKII inhibitor	CaMKII inhibition	
Dezocine	Mixed opioid R partial agonist/antagonist	CaMKII phosphorylation inhibition	
Lidocaine	VGSC blocker		
Anxa1(2–26)	Annexin A1-derived peptide		
Naloxone	Opioid antagonist	NMDAR antagonism, TLR4 antagonism	NMDAR inhibition, TLR4 inhibition
Roscovitine	Cdk5 inhibitor	NMDAR phosphorylation inhibition, mGluR5 phosphorylation inhibition	NMDAR inhibition, mGluR5 inhibition
TDZD-8	Selective GSK-3β inhibitor	NMDAR antagonism, NMDA expression, trafficking inhibition, AMPAR expression inhibition	NMDAR inhibition, AMPAR inhibition
PHA-543613	α7-nAChR selective agonist	NMDAR phosphorylation inhibition, proinflammatory cytokine reduction	NMDAR inhibition, proinflammatory cytokine reduction
PNU-120596	α7-nAChR type II PAM	NMDAR phosphorylation inhibition, proinflammatory cytokine reduction, KCC2 expression enhancement	NMDAR inhibition, proinflammatory cytokine reduction, KCC2 enhancement
N-acetyl-cysteine	Cysteine donor	MMP-9 inhibition and NMDAR phosphorylation inhibition	NMDAR inhibition, proinflammatory cytokine reduction, glial inhibition
LiCl	Selective GSK-3β inhibitor	AMPAR expression inhibition	AMPAR inhibition
Naltrindole	Selective DOR inhibitor	AMPAR antagonism, AMPAR trafficking inhibition	AMPAR inhibition
JWH015	CB2 agonist	Glial inhibition, proinflammatory cytokine production inhibition, CB2 expression enhancement, NMDAR phosphorylation inhibition	Glial inhibition
Minocycline	Microglia inhibitor	Inhibitor of microglia proliferation and proinflammatory cytokine release	Glial inhibition, proinflammatory cytokine inhibition
Maropitant	NK-1 antagonist	NK-1 R antagonism	NK-1 R inhibition
BDNF-sequester TrkB/Fc	BDNF neutraliser	KCC2 expression enhancement	BDNF/trkB-KCC2 signal enhancement
Betulinic acid	Pentacyclic triterpenoid	Malondialdehyde, 3-nitrotyrosine, and proinflammatory cytokine production inhibition	Oxidative stress reduction, proinflammatory cytokine reduction

In fentanyl trials, 8 different interventions were studied (Table 7). 6 of these share the NMDAR inhibition mechanism group that consists of NMDAR block, NMDAR antagonism and CaMKII inhibition. Other mechanisms for OIH attenuation include cyclooxygenase (COX) inhibition by ketorolac and voltage-gated calcium channel (VGCC) inhibition by gabapentin.

**Table 7:** Mechanisms of the interventions in fentanyl trials. Eight interventions’ general class, OIH attenuation mechanism, and mechanism group based on the shared pathway for OIH attenuation.

Intervention	General Class	OIH Attenuation Mechanism	Mechanism Group
Ketamine	NMDAR blocker	NMDAR block	NMDAR inhibition
Magnesium			
BN2572	NMDAR-antagonist	NMDAR antagonism	
Nitrous oxide	NMDAR antagonist and benzodiazepine agonist		
Sevoflurane	Volatile anaesthetic		
KN93	CaMKII inhibitor	CaMKII inhibition	
Ketorolac	COX inhibitor	COX inhibition	COX inhibition
Gabapentin	VGCC inhibitor	VGCC inhibition	VGCC inhibition

The mechanisms of 52 interventions studied in morphine trials are summarised in Table 8. The largest mechanism group is NMDAR inhibition, which consists of seven interventions with NMDAR inhibition mechanism alone and four further interventions with NMDAR inhibition and other potential mechanisms to attenuate OIH. The second-largest mechanism group is sphingosine-1-phosphate receptor (S1PR) inhibition (eight interventions), including antagonists for different S1PR subtypes and S1pr1 silencer. Yet, all of the S1PR inhibition interventions were studied only by Doyle et al. [27]. Other large mechanism groups are opioid receptor inhibition (four interventions), endoplasmic reticulum (ER) stress suppression (four interventions), inhibition of glial cells and proinflammatory cytokine reduction (three interventions), and α2-adrenergic receptor inhibition (three interventions). The general class of olive leaf extract and *Withania somnifera* root extract is marked as “unclear” since they are a mixture of several compounds. However, for both interventions, potential OIH attenuation mechanisms have been identified. Olive leaf extract is thought to work by blocking calcium channels and reducing proinflammatory cytokine levels. *Withania somnifera* root extract is suggested to work via γ-aminobutyric acid (GABA) type A and B receptor agonism, NMDAR antagonism, and DOR antagonism. The mechanisms of Re ginsenoside, Rg1 ginsenoside, and Rb1 ginsenoside were marked as “not known” since their potential mechanisms for OIH attenuation are not yet understood.

**Table 8:** Mechanisms of the interventions in morphine trials. General class of 52 interventions, OIH attenuation mechanism, and mechanism group based on the shared pathway for OIH attenuation.

Intervention	General Class	OIH Attenuation Mechanism	Mechanism Group
Ketamine	NMDAR blocker	NMDAR block	NMDAR inhibition
Magnesium			
Dextromethorphan	NMDAR antagonist	NMDAR antagonism	
MK-801			
D-serine	NMDAR agonist	NMDAR internalisation	
PLGA curcumin	Diarylheptanoid	CaMKII inhibition	
Unformulated curcumin			
CTOP	MOR antagonist	MOR antagonism	Opioid R inhibition
DAMGO			
Methylnaltrexone bromide	Peripherally restricted MOR antagonist	MOR antagonism	
Naltrexone	Opioid antagonist	Excitatory opioid R inhibition	
Withania somnifera root extract	Unclear	GABAAR agonism, GABABR agonism, NMDAR antagonism, DOR antagonism	GABAAR enhancement, GABABR enhancement, NMDAR inhibition, DOR inhibition
Gabapentin	VGCC inhibitor	Adenylyl cyclase, PKC inhibition, NMDAR antagonism	Adenylyl cyclase, PKC inhibition, NMDAR inhibition
Melatonin	Melatonin R agonist	cAMP downregulation, PKC inhibition, NMDAR expression inhibition	cAMP reduction, PKC inhibition, NMDAR inhibition
AEBSF	Serine protease inhibitor	ER stress suppression, NMDAR and PKA phosphorylation inhibition	ER stress suppression, NMDAR and PKA phosphorylation inhibition
4μ8C	IRE1α inhibitor		
TUDCA	ER stress suppressor	ER stress suppression	ER stress suppression
Salubrinal	eIF2α dephosphorylation inhibitor		
Adenovirus-HSP70	HSP70 overexpressor		
Glibenclamide	Potassium channel blocker		
Propentofylline	Phosphodiesterase inhibitor	Glial inhibition and proinflammatory cytokine reduction	Glial inhibition and proinflammatory cytokine reduction
L-732,138	Tachykinin NK1 R antagonist		
MSC	Stem cell		
Olive leaf extract	Unclear	Calcium channel block, proinflammatory cytokine reduction	Calcium channel inhibition, proinflammatory cytokine reduction
Amlodipine	L-type calcium channel blocker	L-type calcium channel block	L-type calcium channel inhibition
Nifedipine	Calcium channel blocker	Morphine-induced corticosterone secretion inhibition	Morphine-induced corticosterone secretion inhibition
Ibuprofen	COX inhibitor	COX inhibition	COX inhibition
Ketorolac	COX inhibitor		
Raf-1 siRNA	Raf-1 siRNA	Raf-1 expression inhibition	CGRP inhibition
PKA siRNA	PKA siRNA	PKA expression inhibition	CGRP inhibition
Ondansetron	5-HT3 R antagonist	5-HT3 R antagonism	5-HT3 R inhibition
Buspirone	5-HT1A R partial agonist	5-HT1A agonism	5-HT1A enhancement
Ceftriaxone	β-lactam antibiotic	GLT-1 expression enhancement	GLT-1 enhancement
Efaroxan	α2 adrenergic R antagonist	α2 adrenergic R antagonism	α2 adrenergic R inhibition
Atipamezole			
Yohimbine			
CLP257	KCC2 enhancer	KCC2 expression enhancement	KCC2 enhancement
CLP290			
PD98059	MEK inhibitor	ERK pathway inhibition	ERK pathway inhibition
GAT211	CB1R PAM	CB1R PAM	CB1R enhancement
W146	S1PR1 antagonist	S1PR antagonism	S1PR inhibition
NIBR-14			
NIBR-15			
Fingolimod			
Ponesimod			
JTE-013	S1PR2 antagonist		
CAY10444	S1PR3 antagonist		
S1pr1 silencer	S1PR expression inhibition	S1pr1 silencer	
SEW2871	S1PR1 agonist	S1PR agonism	S1PR enhancement
Re ginsenoside	Ginsenoside	Not known	Not known
Rg1 ginsenoside			
Rb1 ginsenoside			

## 5. Discussion

### 5.1. Summary of Evidence

This review identified 72 trials that in total investigated 82 different pharmacological interventions for OIH. There were 27 trials on RIH, 14 trials on FIH, and 31 trials on MIH. In the remifentanil trials, 27 different interventions were investigated while the fentanyl trials investigated 9 interventions and the morphine trials investigated 52 interventions. The majority of the interventions (68 out of 82, 83%) were studied in only one trial. Overall, ketamine and gabapentin were the most-investigated interventions. Only four combination interventions were investigated: ketamine and gabapentin, hydrogen-rich saline and Ro 25-6981, PHA-543613 and PNU-120596, and ketamine and KN93. Furthermore, only 17 trials investigated more than one intervention, and only Doyle et al. [27] and Lin et al. [58] investigated more than three interventions. These 17 trials can provide direct comparisons of interventions, although some of the trials slightly varied their experimental methodology between the interventions they tested. Therefore, to compare the effectiveness of most of the interventions in the literature, a network meta-analysis is required. Only 9 of the 82 interventions were found ineffective in attenuating OIH by all tested doses. The Aguado et al. [29] trial was the only remifentanil trial that studied ineffective interventions. These were amitriptyline, minocycline, and maropitant. In morphine trials, ineffective interventions included Rg1 ginsenoside and Rb1 ginsenoside studied by Li et al. [86]; GAT211 studied by Datta et al. [89]; and JTE-013, CAY10444, and SEW2871 studied by Doyle et al. [27]. In fentanyl trials, no ineffective interventions were reported. Hence, 89% of the tested interventions were reported effective, and this high percentage raises a concern for the presence of positive result publication bias in the literature.

The characteristics of each type of opioid trials reflect the opioid’s clinical use and pharmacological properties. Remifentanil is a short-acting opioid with an elimination half-time of 10–20 min and is often used in general anaesthesia [90]. This was mirrored in the preclinical trials, as most of the trials used a plantar incision injury model to mimic surgery, the most common remifentanil administration regimens were 30-min and 60-min infusions, and most of the trials lasted only 2 days. Fentanyl is also mostly used in surgical settings, but may be used to treat chronic pain patients or renal failure patients with severe pain [91]. This is reflected in the preclinical trials as a mixed-use of plantar incision and carrageenan injection injury models as well as a lack of injury model. Although non-nociceptive environmental stress (NNES) is not technically a physiological injury model, it was included in the injury model category of two trials where it was used as stress experienced by patients that may influence the extent to which they develop OIH [26]. Nevertheless, the majority of fentanyl trials (nine trials) did not use any injury model, and hence it is recommended that future fentanyl trials would use a plantar incision injury model to mirror fentanyl’s perioperative use. Moreover, in most of the fentanyl trials, fentanyl was administered via four subcutaneous injections within an hour. This is because fentanyl distributes rapidly from plasma to highly vascular tissues such as the heart, lungs, and the brain, and then redistributes rapidly to muscle and fat—whereas several injections force fentanyl to accumulate to its site of action in the brain [92]. Muscle and fat act as storages for fentanyl and slowly release it back to plasma for elimination, which gives fentanyl a long elimination half-time of 3–8 h. This may explain why most of the fentanyl trials had longer trial lengths (5–8 days) than the remifentanil trials. In all of the fentanyl trials, fentanyl was administered within an hour, but since fentanyl may be used for chronic pain and preclinical trials have demonstrated hyperalgesia after chronic fentanyl administration [93], future intervention trials should include chronic fentanyl administration. Morphine is used for moderate to severe acute and chronic pain and is used, for example, in palliative care and for cancer pain and arthritis [94]. This may explain why the majority of the morphine trials investigated OIH after chronic morphine administration, had long trial lengths (most trials ranged between 5–20 days), and lacked injury models.

Overall, the most common intervention mechanism group was found to be NMDAR inhibition. NMDAR inhibition was the mechanism group of 20 interventions and was one of the mechanisms of 10 other interventions. NMDAR inhibition was also the most common mechanism in each type of opioid trial. This suggests that OIH caused by different opioids can be treated with the same interventions. However, the abundance of trials supporting NMDAR inhibition cannot be used to conclude that the mechanism is necessarily the most effective for OIH caused by one or all of the opioids. The effectiveness of interventions and mechanism groups can only be concluded after a meta-analysis. Other large mechanism groups included S1PR inhibition (eight morphine trials), ER stress suppression (six morphine trials), opioid receptor inhibition (five morphine trials), and proinflammatory cytokine reduction (five remifentanil trials, two morphine trials). It should be noted that when opioid receptor inhibition is used for OIH attenuation, the interventions are not meant to alter opioid analgesia. For example, naltrexone can be given in ultra-low doses [76], and methylnaltrexone bromide is peripherally restricted [53], which allows the analgesia to be preserved.

The findings by Heinl et al. [14] show that RIH occurs via long-term potentiation of synaptic strength in the spinal cord dorsal horn C-fibres via activation of spinal MORs and NMDARs. On the other hand, FIH and MIH occur via an enhancement of synaptic transmission at spinal cord dorsal horn C-fibres via activation of spinal MORs and NMDARs as well as via descending facilitation of C-fibre-evoked field potentials by activation of 5-HT3Rs and extraspinal MORs. These findings suggest that interventions producing NMDAR inhibition are likely to attenuate OIH caused by all opioids, but FIH and MIH may be most effectively targeted with interventions including 5-HT3R inhibition. None of the fentanyl or morphine trials identified investigated combinations of interventions producing NMDAR inhibition and 5-HT3R inhibition. Yet, the Liang et al. [82] morphine trial studied ondansetron, which is a 5-HT3R antagonist, and demonstrated that MIH can be attenuated with a 1 μg intrathecal injection or 2 mg/kg subcutaneous injection of ondansetron. Hence, future fentanyl and morphine trials should investigate whether interventions producing 5-HT3R inhibition may have an additive or synergic effect with interventions producing NMDAR inhibition. Moreover, Roeckel et al. [95] presented an extensive review of the cellular and molecular mechanisms of OIH, which demonstrates that the overall mechanism is a complex process involving multiple pathways. Many of the interventions identified in this scoping review can be connected to the mechanisms they presented such as glial cell activation, production of proinflammatory cytokines, sphingolipid ceramide upregulation, diminished KCC2 action, and neurokinin-1 receptor antagonism. Nevertheless, the mechanism for OIH is still not fully elucidated and the OIH mechanisms of different opioids are not much discussed [11]. The lack of full understanding of OIH mechanisms is also a challenge for OIH intervention research as the interventions are generally planned according to the research on the mechanisms.

Furthermore, it has been shown that the choice of experimental animal model affects the extent to which OIH may be observed, which can also have an effect on the conclusion of OIH intervention trials. It has been acknowledged that there are sex-related differences in the experience of pain [96] and this has been also documented in OIH. For example, in Holtman and Wala’s [97] experiment, MIH was more pronounced in female Sprague–Dawley rats compared to male rats. Hence, it is important to note that in this scoping review the majority of the trials were conducted with male rats or mice and only two trials included female rats. These trials were the Mert et al. trial [38] that investigated the effect of magnesium on FIH with female Wistar rats, and the Doyle et al. trial [27] that included female Sprague-Dawley rats in their MIH and fingolimod experiment. Therefore, when the efficacy of OIH interventions is evaluated in RCTs, it is recommended that a subgroup analysis is conducted to compare the results in women and men. Moreover, the genetic background of the animal model has also been shown to influence the development of OIH. Liang et al. [98] analysed 15 strains of mice and quantified the reduction in mechanical pain threshold after 4 days of morphine administrations. They observed the largest reduction in MRL/MpJ mice (89%) and the smallest reduction in 129/SvlmJ mice (28.5%), which demonstrates how significantly the choice of animal model may impact a trial. The characteristics of the animal models have also been shown to interact with other trial characteristics. For instance, the experiments by Juni et al. [99] provide evidence that the extent of OIH is influenced by the interaction of the sex of the animal and the opioid dose used. They demonstrated that 1.6 mg/kg daily doses of morphine caused MIH in male and female CD-1 mice, but the male’s pain threshold returned to baseline on day 6 while the female’s pain threshold was still significantly different from the baseline on day 12. Yet, when male and female CD-1 mice were given 40 mg/kg daily doses of morphine, both groups’ thresholds returned to baseline on day 12. After all, since it has been well demonstrated that the choice of animal model significantly impacts the extent to which OIH is observed, it raises a question which of the models is most similar to humans and whether any of them is adequately similar. In addition, it could be argued that the perspective of individualised pharmacology may be important in finding the most effective OIH interventions since the sex and genetic background of humans can influence the extent to which one experiences OIH and responds to OIH interventions.

Although the issue of sample size in preclinical trials has not been recognised to be as important as in clinical trials, the number of animals in each study group should be selected using the best available statistical models [100]. If a sample size is too small, a trial may not be able to represent the phenomena it is investigating in its true effect, while choosing an unnecessarily large sample size is a waste of resources and is ethically less justified. The majority of the trials included in this review used 6–12 animals per each study group. In six trials, more than 12 animals were used. Dunbar et al. [44] used the highest number of animals per study group (up to 24). In the trials by Chen et al. [45], Milne et al. [46], and Doyle et al. [27], less than 4-5 animals were included in some of the study groups. Moreover, the size of the study groups was not mentioned in the trials by Dogrul et al. [48] and Li et al. [86]. In the end, most of the trials did not provide an explanation of how a specific number of animals per study group was chosen, which would be good practice in the future.

The choice of QST could also affect the results of a trial. Kang et al. [42] demonstrated that the extent to which OIH is observed may depend on the QST used. They compared von Frey filaments, a radiant heat paw withdrawal test, and an analgesimeter (paw pressure withdrawal test) in male Sprague-Dawley rats that received a 320 μg subcutaneous fentanyl injection. The rats were monitored for 4 days. Using the radiant heat paw withdrawal test, a slight reduction in the pain threshold was observed through the 4-day period but the results were not statistically significant. In contrast, when the pain threshold was monitored using von Frey filaments, a statistically significant reduction was observed in each day of the measurements. On the other hand, pain threshold monitoring with the analgesimeter exhibited a statistically significant reduction on days 2 and 3, while on the 4th day, the pain threshold returned to baseline. Differing results based on the choice of QST have also been observed in intervention experiments. Dunbar et al. [52] tested the effect of ibuprofen on MIH in male Sprague-Dawley rats. The rats received morphine administrations for 5 days and a 10 μL spinal catheter bolus of 1.36 nM, 13.6 nM, or 136 nM ibuprofen on the 5th day. Their pain thresholds were monitored for an hour on the 5th day by recording paw withdrawal in response to radiant heat as well as monitoring several withdrawal behaviours (head shaking, teeth chattering, spontaneous squeaking, jumping, urination, and squeaking) in response to von Frey filaments. Using the radiant heat test, the results demonstrated that the lowest dose was not effective in attenuating OIH, while the results of the highest dose group were similar to the morphine free group. In contrast, none of the intervention groups were different from the placebo group when withdrawal behaviours in response to von Frey filaments were monitored. Furthermore, Cui et al. [33] demonstrated that the body area used for QST can influence the results of a trial. They conducted a plantar incision surgery in male Sprague-Dawley rats under remifentanil anaesthesia and investigated the effect of lidocaine on RIH. Hyperalgesia was quantified using von Frey filaments in the hind paw with the incision (ipsilateral paw) and in the contralateral hind paw. The results indicated that RIH was significantly more pronounced in all of the study groups in the ipsilateral paw compared to the contralateral paw. Yet, several of the trials included in this review that used an injury model failed to mention whether their QST was conducted on the ipsilateral or contralateral paw, which could affect the interventions’ indirect efficacy comparisons. Despite the varying observations from different QSTs and QST methodologies, no reviews were found to evaluate the use of QSTs in preclinical trials.

While this review aimed to analyse trials investigating OIH caused by remifentanil, fentanyl, or morphine, in the screening process it was noted that the search would have identified only two trials conducted with other opioids. These trials were Minville et al. [101] that studied sufentanil-induced hyperalgesia (SIH) and Abreu et al. [102] that studied tramadol-induced hyperalgesia (TIH). Minville et al. [101] investigated the effect of a subcutaneous ketamine injection (1, 10, or 50 mg/kg) on SIH with C57BL/6 male mice that underwent tibial closed fracture surgery that was meant to mimic orthopaedic surgery. The mice were monitored for 7 days and their pain thresholds were measured using von Frey filaments and a hot plate test. The results demonstrated that none of the tested doses of ketamine attenuated SIH when measured using von Frey filaments, but all the tested doses attenuated SIH when measured with a hot plate test. Abreu et al. [102] monitored the effect of 10 mg/kg subcutaneous ketamine injection on TIH in male Wistar rats for 21 days. The rats’ pain thresholds were measured using von Frey filaments and a digital Randall-Selitto device. Ketamine was found to attenuate TIH in both tests. In conclusion, these trials support the argument that OIH caused by all types of opioids can be attenuated via NMDAR inhibition.

### 5.2. Limitations

The focus of this review was to identify pharmacological interventions for OIH, but this perspective itself has limitations. Theoretically, it would be more ideal to avoid opioid use in the first place to avoid the development of OIH. In surgical settings, opioid use is common, but according to Lavand’homme [103], opioid-free anaesthesia can be recommended. In addition, novel multi-functional peptides with MOR agonism and neuropeptide FF receptor antagonism have been developed by Drieu La Rochelle et al. [104] and Zhang et al. [105]. These peptides could provide potent analgesia without hyperalgesia. Furthermore, Comelon et al. [106] and Richebé et al. [107] have shown that adjustments to opioid administration such as more gradual withdrawal or target-controlled infusion could be used to prevent OIH. Recommending non-pharmacological interventions such as exercise or patient education [11] would also have benefits over pharmacological interventions, as using any medication will always have a risk for side effects. Nevertheless, opioids are still used frequently and much more research is needed in each of the OIH prevention and intervention perspectives before they can be taken into clinical practice. Moreover, a review of the numerous preclinical trials conducted on pharmacological OIH interventions is vital for avoiding repetition of similar work and for planning better preclinical trials and RCTs in the future.

The methodology of this scoping review was planned in line with the best available guidelines, yet a few points for improvement can be identified. After the article selection was completed, a couple of potential search items were identified that were not used in the searches. These included for example “pain sensitisation” and “pretreatment”. However, the lack of these search items should not have much effect on missing potential articles, since the references of all eligible trials included were scanned. Since the study selection and data extraction processes were carried out by only one researcher, the possibility for errors could have been reduced by a second reviewer. As discussed, a high likelihood for publication bias in the reviewed literature was identified, and so steps could have been taken to conduct an even wider search. Searches in the grey literature, for example through Google Scholar, could have been carried out. In addition, the analysis could have included relevant abstracts of trials that did not provide a full article and the abstracts of non-English articles.

The results of some of the data extraction groups had to be simplified in order to represent the data in a sensible qualitative way. This mostly concerned the interventions’ effectiveness in each experimental group, opioid regimens, and intervention mechanisms. For example, intervention in an experimental group was categorised as “effective” if a statistical difference to the opioid-only group could be shown at any point with any type of QST. Yet, categorising experimental groups as “effective” or “not effective” oversimplifies the real spectrum of efficacy. This categorisation hides findings such as if an intervention was not found effective with all types of QST that were used, or if an intervention was only minimally effective at a certain time point. Ideally, a meta-analysis of the trials should be conducted, as statistical comparisons between the interventions would allow a more realistic representation of the efficacies. Moreover, opioid regimens were categorised as “acute” if an opioid was administered for less than one day and “chronic” if an opioid was administered for several days. However, the separation between acute and chronic administration is slightly arbitrary and was mostly used to emphasise the different lengths of the regimens. The majority of the trials with an acute regimen administered the opioid within one 1 h and only a few administered the opioid up to 3 h, whereas all of the chronic regimen trials administered the opioid for at least 24 h. When quantifying the efficacies of the interventions, it should be analysed whether the length of opioid use has any effect on the intervention ranking or doses required for OIH attenuation. Furthermore, the OIH attenuation mechanism and the mechanism group of each intervention were categorised based on the authors’ findings and conclusions presented in the eligible articles and no further studies in the literature were explored. Some of the articles investigated the potential mechanism via in vitro experiments or using transgenic animal models, whereas some cited others’ research. In a few articles, the mechanism suggested was based on an educated guess. Hence, the mechanism classification presented may provide a simplified view of the actual mechanisms, and alternative suggestions could be found in additional research.

The long-term objective of reviewing the preclinical trials investigating pharmacological interventions for OIH is to identify the most effective agents that could be used for humans. However, generalising the findings from animals to humans is not straightforward. According to the Leenaars et al. [108] review, preclinical to clinical translational success varies considerably, and the translational success tends to be unpredictable. Additionally, preclinical trials do not incorporate factors such as culture or human psychological phenomena, which is why RCTs should be used to evaluate if the most effective OIH interventions vary in different contexts. Nevertheless, preclinical trials have the strength of having fewer uncontrolled variables compared to trials with humans, which can be beneficial in generalising results across different situations.

### 5.3. Conclusions

In summary, this scoping review aimed to identify and describe all the preclinical trials investigating pharmacological interventions for OIH caused by remifentanil, fentanyl, or morphine as the first step towards evaluating whether the most effective OIH interventions are different for different opioids. Plenty of preclinical trials and interventions were identified. However, many of the interventions were studied in only one trial, which means that the evidence behind each intervention is not very strong. Furthermore, since only a few of the trials can provide direct comparisons of effectiveness, the interventions could be indirectly compared in a meta-analysis to identify the most effective ones for each opioid. In addition, conducting more preclinical trials comparing different interventions is recommended. Additionally, in the current literature, very few combination interventions were investigated, which could be addressed in future trials. NMDAR inhibition was found to be the most-studied mechanism for OIH attenuation for all of the opioids. Yet, the abundance of evidence alone cannot be used to conclude that the mechanism is necessarily the most effective for OIH caused by one or all of the opioids. In the end, these preclinical trials have provided several successful OIH interventions whose effectiveness for clinical use could be tested in RCTs.

## Data Availability

All data generated or analysed during this study are included in this published article and its supplementary information files.

## Supplementary Materials

The following supporting information can be downloaded at: https://www.mdpi.com/article/10.3390/jcm11237060/s1, File S1: Pharmacological Interventions for Opioid Induced Hyperalgesia Data Extraction.

## Abbreviations

5-HT3 R	5-hydroxytryptamine type 3 receptor
5-HT1A R	5-hydroxytryptamine type 1A receptor
AEBSF	4-(2-aminoethyl)benzenesulfonyl fluoride hydrochloride
α7-nAChR	alpha 7-nicotinic acetylcholine receptor
BDNF	brain-derived neurotrophic factor
CaMKII	calcium/calmodulin-dependent protein kinase II
cAMP	cyclic adenosine-monophosphate
CB1	cannabinoid receptor type 1
CB2	cannabinoid receptor type 2
CCI	chronic constriction injury
Cdk5	cyclin dependent kinase 5
CI	carrageenan injection
COX	cyclooxygenase
DOR	delta-opioid receptor
eIF2α	eukaryotic initiation factor-2 alpha
ER	endoplasmic reticulum
ERK	extracellular signal-regulated kinase
FIH	fentanyl-induced hyperalgesia
GABAAR	gamma-aminobutyric acid type A receptor
GABABR	gamma-aminobutyric acid type B receptor
GLT-1	glutamate transporter-1
GSK-3β	glycogen synthase kinase-3 beta
HSP70	heat shock protein 70
HRS	hydrogen-rich saline
i.g.	intragastric
i.p.	intraperitoneal
i.t.	intrathecal
i.v.	intravenous
ICR	Institute of Cancer Research
IRE1α	inositol-requiring transmembrane kinase endoribonuclease-1 alpha
IWP-2	inhibitor of Wnt productions-2
KCC2	potassium-chloride cotransporter
LiCl	lithium chloride
mGluR5	metabotropic glutamate receptor type 5
MEK	mitogen-activated and extracellular signal-regulated kinase
MMP-9	matrix metallopeptidase 9
MSC	mesenchymal stem cell
NAC	N-acetyl-cysteine
NK-1	neurokinin-1
NMDAR	N-methyl-D-aspartate receptor
NNES	non-nociceptive environmental stress
NR2B	N-methyl-D-aspartate receptor subunit 2B
OIH	opioid-induced hyperalgesia
PAM	positive allosteric modulator
PI	plantar incision
PKA	protein kinase A
PKC	protein kinase C
PLGA	polylactic-co-glycolic acid
QST	quantitative sensory test
Raf-1	rapidly accelerated fibrosarcoma 1
RIH	remifentanil-induced hyperalgesia
s.c.	subcutaneous
S1PR	sphingosine-1-phosphate receptor
siRNA	small interfering ribonucleic acid
SIH	sufentanil-induced hyperalgesia
TDZD-8	thiadiazolidinone-8
TIH	tramadol-induced hyperalgesia
TLR4	toll-like receptor 4
TUDCA	tauroursodeoxycholic acid
TrkB/Fc	tropomyosin receptor kinase fragment crystallizable (region)
VGCC	voltage-gated calcium channel
VGSC	voltage-gated sodium channel

## Appendix A. PRISMA-ScR Checklist

**Table A1:** Summary of PRISMA-ScR checklist. The PRISMA-ScR checklist provides subsections for the introduction, methods, results, and discussion that should be followed in scoping reviews.

Main Section	Subsection	Number of the Item in the Review
Title	-	-
Abstract	-	-
Introduction	Rationale	1.
	Objectives	2.
Methods	Registration	N/A
	Protocol	3.1.
	Eligibility criteria	3.2.
	Information sources	3.3.
	Search	3.4.
	Selection of sources of evidence	3.5.
	Data charting	3.6.
	Data items	3.6.
	Critical appraisal of individual sources of evidence	N/A
	Synthesis of results	3.7.
Results	Selection of sources of evidence	4.1.
	Characteristics of sources of evidence	4.2.
	Critical appraisal within sources of evidence	N/A
	Results of individual sources of evidence	4.3.
	Synthesis of results	4.4.
Discussion	Summary of evidence	5.1.
	Limitations	5.2.
	Conclusions	5.3.
Funding	-	N/A

## Appendix B. Search Strategies

**Table A2:** Embase search strategy development (via Ovid). The number of records retrieved for search items in Embase.

	Search Terms	Results
1	opioid induced hyperalgesia/	306
2	opioid induced hyperalgesia.mp.	878
3	opioid-induced hyperalgesia.mp.	878
4	opioid induced hypersensitivity.mp.	7
5	remifentanil induced hyperalgesia.mp.	114
6	fentanyl induced hyperalgesia.mp.	21
7	morphine induced hyperalgesia.mp.	77
8	1 or 2 or 3 or 4 or 5 or 6 or 7	1016
9	Animal research filter by van der Mierden et al. (2021)	7,961,031
10	8 and 9	413
11	drug therapy.mp. or drug therapy/	4,902,242
12	pharmacological intervention.mp.	7773
13	pharmacological treatment.mp.	27,737
14	pharmacotherapy.mp.	53,406
15	rehabilitation.mp. or rehabilitation/	398,934
16	treat*.mp.	9,169,701
17	intervention.mp. or intervention study/or early intervention/	1,108,613
18	prevent*.mp. or prevention/	3,040,390
19	remedy.mp.	16,393
20	acetazolamide.mp. or acetazolamide/	20,761
21	amantadine.mp. or amantadine/	16,993
22	amlodipine.mp. or amlodipine/	28,139
23	buprenorphine/or buprenorphine.mp.	23,199
24	butorphanol/or butorphanol.mp.	5151
25	clonidine/or clonidine.mp.	44,729
26	dextro serine.mp. or dextro serine/or d-serine.mp.	3484
27	dexmedetomidine.mp. or dexmedetomidine/	14,959
28	dextromethorphan/or dextromethorphan.mp.	8385
29	flurbiprofen axetil.mp. or flurbiprofen axetil/	405
30	gabapentin.mp. or gabapentin/	34,854
31	hydrogen-rich saline.mp.	252
32	ketamine.mp. or ketamine/	50,685
33	ketorolac.mp. or ketorolac/	13,261
34	lidocaine.mp. or lidocaine/	82,854
35	magnesium.mp. or magnesium sulfate/or magnesium sulfate.mp. or magnesium sulphate.mp.	168,729
36	methadone/or methadone.mp.	37,154
37	methylnaltrexone bromide.mp. or 17 methylnaltrexone/	1140
38	minocycline.mp. or minocycline/	27,121
39	nalbuphine.mp. or nalbuphine/	3416
40	naloxone/or naloxone.mp.	49,605
41	naltrexone.mp. or naltrexone/	17,475
42	nitrous oxide.mp. or nitrous oxide/	40,446
43	paracetamol.mp. or paracetamol/or acetaminophen.mp.	107,661
44	parecoxib.mp. or parecoxib/	2198
45	pregabalin/or pregabalin.mp.	16,715
46	propofol/or propofol.mp.	63,465
47	propranolol/or propranolol.mp.	98,141
48	11 or 12 or … 48	13,625,596
49	10 and 48	345

**Table A3:** Final Embase search (via Ovid). The number of records retrieved for the disease of interest (1), animal research (2), interventions (3), and their combinations (4).

	Search Terms	Results
1	opioid induced hyperalgesia/or opioid induced hyperalgesia.mp. or opioid-induced hyperalgesia.mp. or opioid induced hypersensitivity.mp. or remifentanil induced hyperalgesia.mp. or fentanyl induced hyperalgesia.mp. or morphine induced hyperalgesia.mp.	1016
2	Animal research filter by van der Mierden et al. (2021)	7,961,031
3	drug therapy.mp. or drug therapy/or pharmacological intervention.mp. or pharmacological treatment.mp. or pharmacotherapy.mp. or rehabilitation.mp. or rehabilitation/or treat*.mp. or intervention.mp. or intervention study/or early intervention/or prevent*.mp. or prevention/or remedy.mp. or acetazolamide.mp. or acetazolamide/or amantadine.mp. or amantadine/or amlodipine.mp. or amlodipine/or buprenorphine/or buprenorphine.mp. or butorphanol/or butorphanol.mp. or clonidine/or clonidine.mp. or dextro serine.mp. or dextro serine/or d-serine.mp. or dexmedetomidine.mp. or dexmedetomidine/or dextromethorphan/or dextromethorphan.mp. or flurbiprofen axetil.mp. or flurbiprofen axetil/or gabapentin.mp. or gabapentin/or hydrogen-rich saline.mp. or ketamine.mp. or ketamine/or ketorolac.mp. or ketorolac/or lidocaine.mp. or lidocaine/or magnesium.mp. or magnesium sulfate/or magnesium sulfate.mp. or magnesium sulphate.mp. or methadone/or methadone.mp. or methylnaltrexone bromide.mp. or 17 methylnaltrexone/or minocycline.mp. or minocycline/or nalbuphine.mp. or nalbuphine/or naloxone/or naloxone.mp. or naltrexone.mp. or naltrexone/or nitrous oxide.mp. or nitrous oxide/or paracetamol.mp. or paracetamol/or parecoxib.mp. or acetaminophen.mp. or parecoxib/or pregabalin/or pregabalin.mp. or propofol/or propofol.mp. or propranolol/or propranolol.mp.	13,625,596
4	1 and 2 and 3	345

**Table A4:** PubMed search strategy development. The number of records retrieved for search items in PubMed.

	Search Terms	Results
1	“opioid induced hyperalgesia” [tw]	558
2	“opioid induced hypersensitivity” [tw]	6
3	“remifentanil induced hyperalgesia” [tw]	94
4	“fentanyl induced hyperalgesia” [tw]	19
5	“morphine induced hyperalgesia” [tw]	63
6	1 or 2 or 3 or 4 or 5	681
7	Animal research filter by van der Mierden et al. (2021)	3,635,995
8	6 and 7	244
9	“drug therapy” [mesh] or “drug therapy” [tw]	3,228,093
10	“pharmacological intervention” [tw]	5440
11	“pharmacological treatment” [tw]	17,511
12	“pharmacotherapy” [tw]	33,828
13	“rehabilitation” [mesh] or “rehabilitation” [tw]	570,257
14	“treat*” [tw]	6,576,870
15	“intervention” [tw] or “early medical intervention” [mesh] or “intervention study” [tw]	718,556
16	“prevent*” [tw]	2,606,090
17	“remedy” [tw]	12,443
18	“Acetazolamide” [Mesh] or “Acetazolamide” [tw]	9586
19	“Amantadine” [Mesh] or “Amantadine” [tw]	7748
20	“Amlodipine” [Mesh] or “Amlodipine” [tw]	5955
21	“Buprenorphine” [Mesh] or “Buprenorphine” [tw]	9051
22	“Butorphanol” [Mesh] or “Butorphanol” [tw]	1761
23	“Clonidine” [Mesh] or “Clonidine” [tw]	18,563
24	“Dextro serine” [tw] or “D serine” [tw]	2184
25	“Dexmedetomidine” [Mesh] or “Dexmedetomidine” [tw]	7487
26	“Dextromethorphan” [Mesh] or “Dextromethorphan” [tw]	3035
27	“Flurbiprofen axetil” [tw]	137
28	“Gabapentin” [Mesh] or “Gabapentin” [tw]	7559
29	“Hydrogen rich saline” [tw]	216
30	“Ketamine” [Mesh] or “Ketamine” [tw]	22,022
31	“Ketorolac” [Mesh] or “Ketorolac” [tw]	3460
32	“Lidocaine” [Mesh] or “Lidocaine” [tw]	33,864
33	“Magnesium” [Mesh] or “Magnesium Sulfate” [Mesh] or “magnesium” [tw] or “magnesium sulfate” [tw] or “magnesium sulphate” [tw]	113,626
34	“Methadone” [Mesh] or “Methadone” [tw]	17,983
35	“Methylnaltrexone bromide” [tw] or “17 methylnaltrexone” [tw]	29
36	“Minocycline” [Mesh] or “Minocycline” [tw]	9586
37	“Nalbuphine” [Mesh] or “Nalbuphine” [tw]	1098
38	“Naloxone” [Mesh] or “Naloxone” [tw]	35,000
39	“Naltrexone” [Mesh] or “Naltrexone” [tw]	10,726
40	“Nitrous Oxide” [Mesh] or “Nitrous Oxide” [tw]	22,788
41	“Acetaminophen” [Mesh] or “Acetaminophen” [tw] or “paracetamol” [tw]	31,289
42	“Parecoxib” [tw]	640
43	“Pregabalin” [Mesh] or “Pregabalin” [tw]	4190
44	“Propofol” [Mesh] or “Propofol” [tw]	24,145
45	“Propranolol” [Mesh] or “Propranolol” [tw]	45,817
46	9 or 10 or … 45	10,427,552
47	8 and 46	211

**Table A5:** Final PubMed search. The number of records retrieved for the disease of interest (1), animal research (2), interventions (3), and their combinations (4).

	Search Terms	Results
1	“opioid induced hyperalgesia” [tw] or “opioid induced hypersensitivity” [tw] or “remifentanil induced hyperalgesia” [tw] or “fentanyl induced hyperalgesia” [tw] or “morphine induced hyperalgesia” [tw]	681
2	Animal research filter by van der Mierden et al. (2021)	3,635,995
3	“drug therapy” [mesh] or “drug therapy” [tw] or “pharmacological intervention” [tw] or “pharmacological treatment” [tw] or “pharmacotherapy” [tw] or “rehabilitation” [mesh] or “rehabilitation” [tw] or “treat*” [tw] or “intervention” [tw] or “early medical intervention” [mesh] or “intervention study” [tw] or “prevent*” [tw] or “remedy” [tw] or “acetazolamide” [mesh] or “acetazolamide” [tw] or “Amantadine” [Mesh] or “Amantadine” [tw] or “Amlodipine” [Mesh] or “Amlodipine” [tw] or “Buprenorphine” [Mesh] or “Buprenorphine” [tw] or “Butorphanol” [Mesh] or “Butorphanol” [tw] or “Clonidine” [Mesh] or “Clonidine” [tw] or “Dextro serine” [tw] or “D serine” [tw] or “Dexmedetomidine” [Mesh] or “Dexmedetomidine” [tw] or “Dextromethorphan” [Mesh] or “Dextromethorphan” [tw] or “Flurbiprofen axetil” [tw] or “Gabapentin” [Mesh] or “Gabapentin” [tw] or “Hydrogen rich saline” [tw] or “Ketamine” [Mesh] or “Ketamine” [tw] or “Ketorolac” [Mesh] or “Ketorolac” [tw] or “Lidocaine” [Mesh] or “Lidocaine” [tw] or “Magnesium” [Mesh] or “Magnesium Sulfate” [Mesh] or “magnesium” [tw] or “magnesium sulfate” [tw] or “magnesium sulphate” [tw] or “Methadone” [Mesh] or “Methadone” [tw] or “Methylnaltrexone bromide” [tw] or “Minocycline” [Mesh] or “Minocycline” [tw] or “Nalbuphine” [Mesh] or “Nalbuphine” [tw] or “Naloxone” [Mesh] or “Naloxone” [tw] or “Naltrexone” [Mesh] or “Naltrexone” [tw] or “Nitrous Oxide” [Mesh] or “Nitrous Oxide” [tw] or “Acetaminophen” [Mesh] or “Acetaminophen” [tw] or “paracetamol” [tw] or “Parecoxib” [tw] or “Pregabalin” [Mesh] or “Pregabalin” [tw] or “Propofol” [Mesh] or “Propofol” [tw] or “Propranolol” [Mesh] or “Propranolol” [tw]	10,427,552
4	1 and 2 and 3	211

**Table A6:** Web of Science search strategy development (via Clarivate). The number of records retrieved for search items in Web of Science.

	Search Terms	Results
1	ts = (“opioid induced hyperalgesia”)	779
2	ts = (“opioid induced hypersensitivity”)	6
3	ts = (“remifentanil induced hyperalgesia”)	94
4	ts = (“fentanyl induced hyperalgesia”)	18
5	ts = (“morphine induced hyperalgesia”)	62
6	1 or 2 or 3 or 4 or 5	861
7	Animal research filter by van der Mierden et al. (2021)	9,810,826
8	6 and 7	439
9	ts = (“drug therapy”)	39,876
10	ts = (“pharmacological intervention”)	5148
11	ts = (“pharmacological treatment”)	18,485
12	ts = (“pharmacotherapy”)	35,666
13	ts = (“rehabilitation”)	245,741
14	ts = (“treat*”)	6,832,946
15	ts = (“intervention” or “intervention study” or “early intervention”)	858,721
16	ts = (“prevent*”)	1,962,441
17	ts = (“remedy”)	29,394
18	ts = (“acetazolamide”)	7226
19	ts = (“amantadine”)	5035
20	ts = (“amlodipine”)	7923
21	ts = (“buprenorphine”)	10,932
22	ts = (“butorphanol”)	2492
23	ts = (“clonidine”)	15,979
24	ts = (“dextro serine” or “d-serine”)	3664
25	ts = (“dexmedetomidine”)	9172
26	ts = (“dextromethorphan”)	3747
27	ts = (“flurbiprofen axetil”)	128
28	ts = (“gabapentin”)	9611
29	ts = (“hydrogen-rich saline”)	232
30	ts = (“ketamine”)	25094
31	ts = (“ketorolac”)	4474
32	ts = (“lidocaine”)	26513
33	ts = (“magnesium” or “magnesium sulfate” or “magnesium sulphate”)	198,241
34	ts = (“methadone”)	19,746
35	ts = (“methylnaltrexone bromide” or “17 methylnaltrexone”)	26
36	ts = (“minocycline”)	8298
37	ts = (“nalbuphine”)	1164
38	ts = (“naloxone”)	22,357
39	ts = (“naltrexone”)	9675
40	ts = (“nitrous oxide”)	36,654
41	ts = (“paracetamol” or “acetaminophen”)	37,680
42	ts = (“parecoxib”)	802
43	ts = (“pregabalin”)	5451
44	ts = (“propofol”)	29,558
45	ts = (“propranolol”)	29,323
46	9 or 10 … or 45	9,247,720
47	8 and 46	348

**Table A7:** Final Web of Science search (via Clarivate). The number of records retrieved for the disease of interest (1), animal research (2), interventions (3), and their combinations (4).

	Search Terms	Results
1	ts = (“opioid induced hyperalgesia” or “opioid induced hypersensitivity” or “remifentanil induced hyperalgesia” or “fentanyl induced hyperalgesia” or “morphine induced hyperalgesia”)	861
2	Animal research filter by van der Mierden et al. (2021)	9,810,826
3	ts = (“drug therapy” or “pharmacological intervention” or “pharmacological treatment” or “pharmacotherapy” or “rehabilitation” or “rehabilitation” or “treat*” or “intervention” or “intervention study” or “early intervention” or “prevent*” or “remedy” or “acetazolamide” or “amantadine” or “amlodipine” or “buprenorphine” or “butorphanol” or “clonidine” or “dextro serine” or “d-serine” or “dexmedetomidine” or “dextromethorphan” or “flurbiprofen axetil” or “gabapentin” or “hydrogen-rich saline” or “ketamine” or “ketorolac” or “lidocaine” or “magnesium” or “magnesium sulfate” or “magnesium sulphate” or “methadone” or “methylnaltrexone bromide” or “17 methylnaltrexone” or “minocycline” or “nalbuphine” or “naloxone” or “naltrexone” or “nitrous oxide” or “paracetamol” or “acetaminophen” or “parecoxib” or “pregabalin” or “propofol” or “propranolol”)	9,247,720
4	1 and 2 and 3	348
